# Effect of *Lactobacillus paracasei* LK01 on Growth Performance, Antioxidant Capacity, Immunity, Intestinal Health, and Serum Biochemical Indices in Broilers

**DOI:** 10.3390/ani14233474

**Published:** 2024-12-01

**Authors:** Weixin Liu, Hong Cheng, Hao Zhang, Guozhen Liu, Xinyu Yin, Cheng Zhang, Runsheng Jiang, Zaigui Wang, Xiaoling Ding

**Affiliations:** 1 College of Animal Science and Technology, Anhui Agricultural University, Hefei 230031, China; 22720181@ahau.edu.cn (W.L.); 23720314@ahau.edu.cn (H.C.); 23720311@stu.ahau.edu.cn (H.Z.); ddskl624@163.com (G.L.); 464583269@stu.ahau.edu.cn (X.Y.); cheng20050502@126.com (C.Z.); jiangrunshen@ahau.edu.cn (R.J.); 2 College of Life Science and Technology, Anhui Agricultural University, Hefei 230031, China; wangzaigui2013@163.com

**Keywords:** *L. paracasei*, broiler, growth performance, serum biochemical indices, serum antioxidants, immune indices, intestinal morphology, intestinal enzyme activities, cecum flora

## Abstract

This study aimed to investigate the effects of adding *L. paracasei* LK01 to the diet on the growth performance, antioxidant capacity, immunity, intestinal health, and serum biochemical indicators of broilers. This study selected 1080 one-day-old broiler chickens with similar body weight, and randomly divided them into six groups, with six replicates in each group and 30 chicks in each replicate. The chicks were fed (1) the basal diet (CON), (2) the basal diet with 10^6^ CFU/kg *L. paracasei* LK01(T1), (3) the basal diet with 10^7^ CFU/kg *L. paracasei* LK01(T2), (4) the basal diet with 10^8^ CFU/kg *L. paracasei* LK01(T3), (5) the basal diet with 10^9^ CFU/kg *L. paracasei* LK01(T4), and (6) the basal diet with 10^10^ CFU/kg *L. paracasei* LK01(T5). The experiment lasted for 42 days. In this study, compared with the CON group, the diet supplemented with *L. paracasei* significantly increased body weight from 1 to 21 days (*p* < 0.05). In addition, the 10^6^ CFU/kg *L. paracasei* LK01 group significantly reduced the activity of glutamic oxaloacetic transaminase and triglyceride levels; the 10^7^ CFU/kg,10^8^ CFU/kg, and 10^9^ CFU/kg *L. paracasei* LK01 groups also reduced serum uric acid and total cholesterol levels (*p* < 0.05). The experimental groups all had lower serum levels of malondialdehyde and interleukin-1β (*p* < 0.01). Except for the 10^6^ CFU/kg group, all experimental groups had significantly lower tumor necrosis factor-α, and the 10^6^ and 10^7^ CFU/kg groups had higher immunoglobulin M levels (*p* < 0.05). In addition, the 10^6^ CFU/kg group significantly reduced the depth of the ileocecal crypts and increased the villus-to-crypt ratio (V/C) of the jejunum and ileum. In addition, dietary supplementation with *L. paracasei* LK01 did not change the α diversity of the microbial community in the cecum, but significantly increased the proportion of *Bacteroides* (phylum) (*p* < 0.05). The 10^6^ CFU/kg group also significantly increased the abundance of beneficial bacteria such as *Ruminococcaceae* (genus), *Lachnospiraceae* (genus), and *Faecalibacterium* (genus) (*p* < 0.05). In summary, this study revealed that adding 10^6^ CFU/kg of *L. paracasei* LKO1 to broiler diets can improve their production performance, serum biochemical indicators, antioxidant, and immune capabilities, as well as cecal flora.

## Simple Summary

*Lactobacillus paracasei* is widely used in food production as an excellent strain, but its application in livestock production is less reported. Therefore, this study investigated the effects of *L. paracasei* LK01 on growth performance, antioxidant capacity, immunity, intestinal health, and serum biochemical indices of broilers, and whether Lactobacillus paracasei LK01 has the potential to be used as a dietary supplement for broilers. The results showed that the addition of *L. paracasei* to the diet could improve the production performance of broilers, improve the serum biochemical indicators of broilers, improve the antioxidant and immune capabilities of broilers, and optimize the cecal flora. The appropriate level of *L. paracasei* LK01 added to the broiler diet was 10^6^ CFU/kg.

## 1. Introduction

Chicken meat is popular worldwide due to its cost-effectiveness, protein richness, and ease of digestion, and its production scale is increasing year by year [1]. However, as broilers are also sensitive to environmental changes, their body temperature regulation ability is not perfect, especially during the early growth stage; their immune systems are also not fully developed, making them vulnerable to stress, which leads to increased morbidity and mortality [2]. Antibiotics significantly improved the efficiency and economic benefits of farming in the early days due to their ability to promote animal growth, enhance feed conversion rates, and prevent and treat diseases [3]. With increasing concerns about antibiotic resistance and rising consumer demand for antibiotic-free products, the research and application of alternatives to antibiotics in animal feed has become a hotspot [4]. Therefore, many green functional alternatives, such as probiotics, prebiotics, antimicrobial peptides, active enzymes, essential oils, and polysaccharides of plant origin, are receiving more and more attention [5].

*Lactobacillus species* have been used as one of the potential alternatives to antibiotics in animal husbandry due to their characteristics of maintaining the balance of animal intestinal flora, promoting nutrient absorption, and improving the immunity of the animal organism, and their application prospects will become more and more extensive. *L. paracasei* is a member of the *Lactobacillus* genus with great potential as a probiotic [6]. It is widely distributed in nature and can be isolated from the human gut, oral cavity, and various fermented foods [7,8,9]. Several studies have shown that L. paracasei also performs well in terms of antibacterial [10,11,12], antioxidant [13,14,15,16], and cholesterol-lowering [17,18,19] effects; maintaining intestinal flora balance [20,21,22,23]; and regulating body immunity [24,25,26,27,28,29]. However, there are currently not many reports on the application of *L. paracasei* in livestock production.

Therefore, our study aimed to investigate the effects of adding *L. paracasei* LK01 to the diet on the growth performance, serum biochemical indicators, immune and antioxidant indicators, inflammatory factor indicators, intestinal morphology, and digestive enzyme activity, and cecal flora of broilers. The optimal addition amount of *L. paracasei* LK01 as a dietary supplement for broilers was also explored.

## 2. Materials and Methods

### 2.1. Bacterial Strain

*L. paracasei* LK01 was isolated from ryegrass fermentation broth by selective deMan Rogosa Sharpe (MRS) medium in our laboratory. After 24 h of incubation at 37 °C in an anaerobic environment in sterile MRS liquid medium, different concentrations of *L. paracasei* LK01 bacterial liquid were prepared by serial dilution and plate counting. According to the research of Liu et al. [30], freeze-dried Lactobacillus paracasei LK01 powder containing 10^6^, 10^7^, 10^8^, 10^9^, and 10^10^ CFU/g viable bacteria was prepared. Lyophilized powder of *L. paracasei* LK01 at different concentrations was added to the basal diet at a concentration of 1 g/kg to produce experimental diets containing different concentrations of *L. paracasei* LK01. (Rye grass fermentation broth was provided by Suzhou Taikang Animal Husbandry Co., Ltd., Zhangjiagang, China)

### 2.2. Birds, Housing, Diets, and Experimental Design

A total of 1080 one-day-old yellow-feathered broilers were randomly divided into 6 groups, with 6 replicates in each group and 30 chicks in each replicate (1/2 male and 1/2 female). The chicks were fed (1) basal diet (CON), (2) basal diet supplemented with 10^6^ CFU/kg *L. paracasei* LK01 (T1), (3) basal diet supplemented with 10^7^ CFU/kg *L. paracasei* LK01 (T2), (4) basal diet supplemented with 10^8^ CFU/kg *L. paracasei* LK01 (T3), (5) basal diet supplemented with 10^9^ CFU/kg *L. paracasei* LK01 (T4), and (6) basal diet supplemented with 10^10^ CFU/kg *L. paracasei* LK01 (T5). The experiment lasted for 42 days. The experiment was conducted at the experimental farm of Muzhi Poultry Company in Anhui Province, China. Prior to the experiment, poultry in each replicate were kept in individual pens, each of which was thoroughly cleaned and sterilized. All pens were maintained under the same feeding conditions and the animals had free access to clean water and feed. The experiment was approved by the Experimental Animal Management and Animal Ethics Committee of the College of Animal Science and Technology, Anhui Agricultural University (No. SYDW-P20190600601). The basal diets were formulated in accordance with the Agricultural Industry Standard of the People’s Republic of China—Chicken Feed (NY_T33-2004) and were modified according to production practices. The dietary and nutrient composition of the feeds provided are shown in Table 1 and Table 2 (pellets).

### 2.3. Sample Collection

At 42 days of age, one experimental broiler roughly matching the mean body weight was selected from each replicate (each group consisted of 6 chickens) for sample collection after 12 h of fasting. Blood samples were collected through wing veins and then centrifuged at 3000 rpm for 10 min at 4 °C for determination of serum antioxidant indices. In addition, 2–3 cm of tissue was collected from the duodenum, jejunum, and ileum of each chicken and fixed in 4% paraformaldehyde for analysis of mucosal morphology. Finally, the cecum contents of the animals were collected, placed in 2 mL freezing tubes, and stored in liquid nitrogen for microbial 16S rRNA gene sequencing.

### 2.4. Growth Performance

Feed intake was recorded daily to determine average daily feed intake (ADFI), calculated as follows: ADFI = (feed intake during test − feed intake remaining during test)/number of days in test. After three weeks of testing, weekly weights were weighed and recorded to determine average daily gain (ADG), calculated as follows: ADG = (final weight of test − initial weight of test)/number of days of testing. Feeder gain ratio (FCR) was calculated as ADFI/ADG.

### 2.5. Serum Parameter Analysis

In this study, various serum biomarkers were measured using a Myriad BS-380 automated biochemistry analyzer (Shenzhen Myriad Biomedical Electronics Co., Ltd., Shenzhen, China) and assay kits from Nanjing Jiancheng Bioengineering Institute (Nanjing, China). These included the activities of alanine aminotransferase (AST), alanine aminotransferase (ALT), alkaline phosphatase (ALP), as well as total protein (TP), albumin (ALB), uric acid (UA), total cholesterol (TC), triglycerides (TG), serum calcium (Ca), and serum phosphorus (P).

### 2.6. Serum Levels, Antioxidant Markers, and Intestinal Digestive Enzymes

The serum levels of immunoglobulin A (IgA), immunoglobulin G (IgG), immunoglobulin M (IgM), and the concentrations of inflammatory factors such as interleukin-1β (IL-1β), interleukin-2 (IL-2), interleukin-6 (IL-6), and tumor necrosis factor (TNF- α) were determined by enzyme immunoassay. The activities of antioxidant-related enzymes such as total superoxide dismutase (T-SOD), glutathione peroxidase (GSH/Px), total antioxidant capacity (T-AOC), malondialdehyde (MDA), and digestive enzyme activities of small intestinal contents (duodenum, jejunum, and ileum) of broilers were determined using specific kits. All commercial kits were purchased from Nanjing Jianjian Bioengineering Institute, Nanjing, China (http://www.njjcbio.com/). Assays were performed according to the manufacturer’s instructions. The information of all kits is shown in Appendix A.

### 2.7. Intestinal Tissue Morphology

Duodenal, jejunal, and ileal tissue specimens were preserved in 4% formaldehyde solution for fixation and paraffin embedding, and the tissues were dehydrated and cleared, sectioned, and stained with hematoxylin and eosin. Measurements of the small intestine villi and crypts were performed using the high-definition LEICA imaging system (version DFC290, Heilbrugger, Switzerland). Chorionic height and corresponding crypt depth were measured on straight and relatively intact chorionic villi, chorionic height was measured from the crypt–chorionic junction to the tip of the chorion, and crypt depth was measured from the base of the crypt to the crypt–chorionic junction. The villus/crypt ratio was calculated by dividing the villus height by the crypt depth (V/C).

### 2.8. 16S rRNA Sequencing and Cecum Microbiota Analysis

16S rRNA sequencing was performed on cecum contents from slaughtered animals. DNA extraction was performed using the E.Z.N.A.^®^ Soil DNA Kit according to the manufacturer’s experimental protocols; DNA purity and concentration were assayed using a microspectrophotometer (NanoDrop2000, Thermo Fisher Scientific, Waltham, MA, USA); the V3-V4 variable region of the 16S rRNA gene was amplified using universal primers 338F and 806R with barcodes; and PCR products were characterized. The PCR products were identified, purified, and quantified; the library was constructed using a NEXTFLEX Rapid DNA-Seq Kit, quantified and verified by Qubit, and sequenced using Illumina’s Miseq PE300/NovaSeq PE250 platform. The raw reads were demultiplexed, the raw sequenced sequences were quality-controlled using fastp [31] (https://github.com/OpenGene/fastp, version 0.20.0, accessed on 29 September 2023) software, and the reads were assembled using FLASH [32] (http://www.cbcb.umd.edu/software/flash, version 1.2.7, accessed on 8 October 2023) software to assemble the read segments. All samples were subjected to OTU clustering of sequences based on 97% [33,34] similarity using UPARSE software [33] (http://drive5.com/uparse/, version 7.1, accessed on 24 October 2023). Sequences within the OTU were annotated for species based on the Greengenes database. Partial least squares discriminant analysis (PLS-DA) was performed on all samples using SIMCA-P v.14.1 software (Umetrics, Umea, Sweden). Finally, differences in species composition between samples were analyzed for alpha and beta diversity. Linear discriminant analysis (LDA) effect size (LEfSe) analyses were performed to identify bacterial taxa rich in variation based on LDA scores > 2.0. The 16S rRNA sequencing data for all the samples were deposited into the NCBI Sequence Read Archive (SRA) under accession number PRJNA1174407 (https://www.ncbi.nlm.nih.gov/bioproject/PRJNA1174407, accessed on 18 October 2024).

### 2.9. Statistics and Analysis of Data

Statistical analysis and result presentation utilized GraphPad Prism version 8 (GraphPad Software, Inc., San Diego, CA, USA). One-way analysis of variance (ANOVA) was used to analyze the experimental results, and the Tukey test was used to compare the significance of multiple data. The results are expressed as mean ± standard deviation. Statistical significance was defined as a *p* value of <0.05.

## 3. Results

### 3.1. Growth Performance

The growth performance of broilers is summarized in Table 3. Compared with the CON group, the BW of broilers in all experimental groups was significantly higher at 21 days (*p* < 0.01); at 35 days, the BW of broilers in T1 and T2 groups was significantly higher (*p* < 0.05). Interestingly, we also found that the BW of all experimental groups increased at 42 days, but the difference was not statistically significant (*p* > 0.05). Compared to the CON group, the FCR of broilers in test groups T1, T2, and T3 was significantly lower *(p* < 0.02) at 28–35 d.

### 3.2. Serum Physiological and Biochemical Indexes

As can be seen from Table 4, compared with the CON group, the serum AST activity and serum TG levels in the T1 group were significantly lower (*p* < 0.05); the serum UA, TC, and TG levels in the T2 group were significantly lower (*p* < 0.01); the serum ALT and AST activities, serum UA, and TC levels in the T3 group were significantly lower (*p* < 0.05); and the serum UA and TC levels in the T4 group were significantly lower (*p* < 0.01).

### 3.3. Serum Immune and Antioxidant Indices

As can be seen from Table 5, compared with the CON group, serum IgM was significantly elevated in both the T1 and T2 groups (*p* < 0.05); in terms of the MDA indicator, the experimental groups were significantly lower (*p* < 0.01). The IL-1 β index was significantly higher in groups T1, T2, T3, and T4 (*p* < 0.01); the TNF-α index was significantly lower in groups T2, T3, T4, and T5 (*p* < 0.05).

### 3.4. Intestinal Tissue Morphology

As can be seen from Table 6, compared with the CON group, the duodenal crypt depth in the T3 group was significantly reduced (*p* < 0.05); the jejunal villus-to-crypt ratio (V/C) in the T1 group was significantly increased (*p* < 0.05); the crypt depth of the ileum was significantly reduced in groups T1, T2, and T4 (*p* < 0.05); and the crypt-to-villus ratio of the ileum was significantly increased in group T1 (*p* < 0.05).

### 3.5. Intestinal Digestive Enzyme Activities

As can be seen from Table 7, compared with the CON group, the protease activity of the duodenum, jejunum, and ileum in the T1 group was significantly increased (*p* < 0.05); in addition, the protease activity of the duodenum and jejunum in the T5 group was significantly increased (*p* < 0.05).

### 3.6. Gut Microbiota

**Figure 1:**
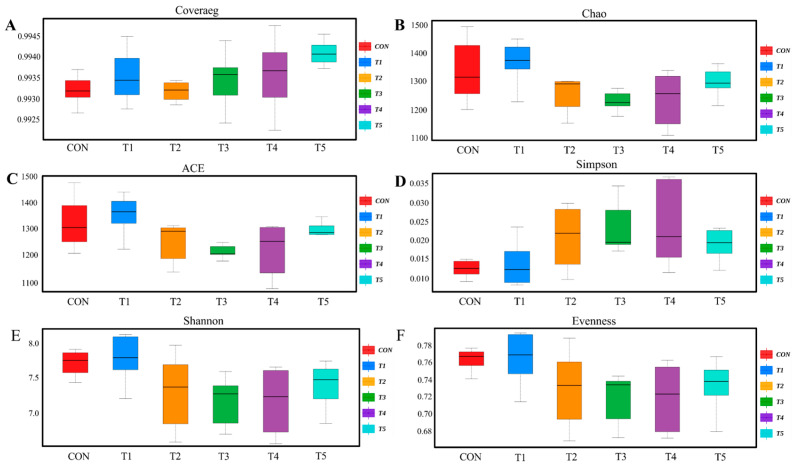
Alpha diversity analysis of the cecal microbiota of broilers. (**A**) Coverage Indexes; (**B**) Chao1 Indexes; (**C**) ACE Indexes; (**D**) Simpson Indexes; (**E**) Shannon Indexes; (**F**) Evenness Indexes.

We analyzed changes in the cecal flora of broilers. Figure 1A demonstrated that all samples achieved coverage indices above 99.0%, indicating comprehensive species diversity and community structure. The alpha diversity index (Chao, Ace, Simpson, Shannon, Evenness) did not significantly differ among groups (Figure 1, *p* > 0.05).

**Figure 2:**
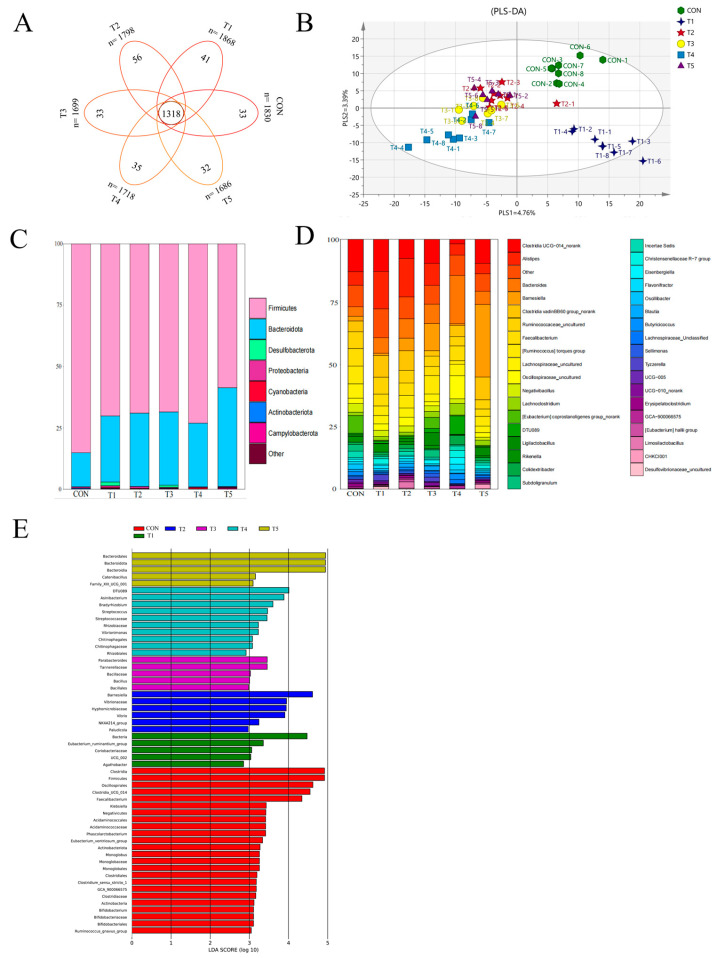
(**A**) OTU Venn diagram; (**B**) PLS-DA score scatter plot; (**C**) taxonomic composition of the cecal microbiota at the phylum level; (**D**) taxonomic composition and relative abundance of the cecal microbiota at the genus level; (**E**) histogram of LDA scores for taxonomic biomarkers identified by LEfSe. LDA scores (log 10) > 2 indicate enriched taxa in cases.

As shown in Figure 2, supplementation with *L. paracasei* LK01 affected the structure of the cecum microbiota in broilers. According to the Venn diagram, the total number of operational taxonomic units (OTUs) was 10,599, and the six groups shared 1318 OTUs (Figure 2A). The PLS-DA plot completely separated the two groups, with axes 1 and 2 explaining 4.76% and 3.39% of the total variation, respectively (Figure 2B). A total of 18 phyla and 304 genera were detected in all samples. At the phylum level, we plotted the top eight dominant bacterial phyla (Figure 2C). Each group mainly detected *Firmicutes*, *Bacteroidetes*, *Proteobacteria*, *Cyanobacteria*, and *Desulfobacterota*. Among them, *Firmicutes* and *Bacteroidetes* are the dominant phyla. Compared with the CON group, the proportion of the *Bacteroidetes* in the experimental group was significantly higher (*p* < 0.05), and the ratio of *Firmicutes* to *Bacteroidetes* (F/B) was significantly lower (*p* < 0.05). In addition, the T1 group also significantly increased the *Cyanobacteria* (*p* < 0.01) and significantly decreased the *Desulfobacterota* (*p* < 0.05). On the genus level, we mapped the top 36 dominant bacterial genera (Figure 2D). The main flora detected in each group included *Clostridia* UCG-014, *Alistipes*, *Bacteroides*, *Clostridia vadinBB60*, *Faecalibacterium*, *Ruminococcaceae, Lachnospiraceae*, and *Negativibacillus*. Among them, *Clostridia*UCG-014 and *Alistipes* are dominant bacteria. Compared with the CON group, the T1 group significantly increased the abundance of *Ruminococcaceae*, *Lachnospiraceae*, and *Faecalibacterium* (*p* < 0.05). The T2 group significantly increased the proportion of *Clostridia vadinBB60* (*p* < 0.01), and the T3 group significantly increased the proportion of *Clostridia* (*p* < 0.05). In addition, the effect size measurement (LEfSe) analysis identified biomarkers with a linear discriminant analysis (LDA) score greater than 2 (Figure 2E).

## 4. Discussion

In this study, we explored the effects of adding *L. paracasei* as a probiotic feed supplement to broilers using formulations with different concentrations of a single-strain product (*L. paracasei* LK01).

Several studies have demonstrated that lactobacilli species as dietary supplements have an enhancing effect on broiler growth performance [6,19,33]. However, the growth effect varies due to many factors such as the source of the strain, the vitality and concentration of the bacteria used, the administration method, and experimental conditions [19,34,35]. A previous study found that when broilers are fed a mixture of *Lactobacillus plantarum* and *Lactobacillus rhamnosus*, their ADG begins to be significantly higher than that of the control group after 2 weeks [36]. Kalavathy et al. [37] found that dietary supplementation with 1% of a 12-strain lactic acid bacteria mixture increased the ADG of broilers from 1 to 42 days of age compared to the control group. Peng et al. [38] showed that supplementing the broiler diet with 2 × 10^9^ CFU/kg *Lactobacillus plantarum* B1 can significantly increase ADG from 1 to 42 days compared to the control group. Another study found that the body weight of 1-day-old male broiler chickens fed a probiotic combination of 2 × 10^6^ CFU/g brewer’s yeast and 1 × 10^7^ CFU/g fermented lactobacillus was higher than that of the control group on the 21st day [39]. This is similar to our research results, which found that all experimental groups increased the body weight of broilers from 0 to 21 days, and that 10^6^ CFU/kg group had the best growth effect. In addition, the FCR of the 10^6^, 10^7^, and 10^8^ CFU/kg groups was significantly reduced during the period from 28 to 35 days.

Serum biochemical indices are important indicators of nutritional metabolism and stress status of the body. In the process of hepatotoxicity, damaged hepatocytes release the liver-specific enzymes ALT and AST into the bloodstream, which then leads to elevation of these two enzymes in the serum [40]. Yilmaz et al. [41] found that sustained chronic heat stress increased serum TG and TC as well as ALT and AST activities and decreased TP and ALB levels. Dietary supplementation of broiler chickens with a variety of *Lactobacillus* strains of synbiotics was found to significantly reduce serum AST activity [42]. Another study found that dietary supplementation with a mixture of *Bacillus licheniformis* and *Bacillus coagulans* significantly reduced serum ALT and AST activities and UA levels [43]. This is similar to our findings, which showed that serum AST levels were significantly reduced in groups 10^6^, 10^8^, and 10^10^ compared to the CON group; serum ALT levels were also significantly reduced in group 10^8^. In addition, UA levels of uric acid were lower than those in the CON group in all experimental groups, and the difference was significant in groups 10^7^, 10^8^, and 10^9^, whereas UA is a nitrogenous excretion product of protein metabolism in poultry, and its serum level is also a direct response to metabolic stress in the kidney [44,45]. Therefore, our results indicate that dietary supplementation with *L. paracasei* LK01 may have a certain effect on improving the liver function of broilers and may reduce serum non-protein nitrogen (UA) to reduce the stress on the kidneys in poultry.

In addition, we found that except for the 10^10^ CFU/kg group, the TC levels of all experimental groups were significantly lower than those of the CON group, and the TG levels of the 10^6^ and 10^7^ groups were significantly lower than those of the CON group. This is similar to the results of several other studies, which found that supplementing the diet of broilers with a 0.1% lactic acid bacteria mixture significantly reduced serum TC and TG levels [46]. Shokryazdan et al. [47] found that dietary supplementation of broilers with 0.5 or 1 g/kg of a mixture of Lactobacillus salivarius both resulted in significantly lower serum TC and TG concentrations in broilers. Elleithy et al. [43] found that supplementing the diet of broiler chickens with a mixture of *Bacillus licheniformis* and *Bacillus coagulans* significantly reduced serum TC levels. Another study showed that dietary supplementation of broiler chickens with *Bacillus amyloliquefaciens* significantly reduced serum TG and TC levels [48]. In addition, our results showed that the ALP and TP levels in all experimental groups were slightly higher than those in the control group, but the difference was not significant. ALP is not only a biomarker of the hepatobiliary system; it is also involved in bone formation [40]. In the event of tissue damage, TP can be used as a raw material to repair and maintain the body’s metabolism [49]. The levels of TC and TG in the blood serum are often used as important indicators of the body’s lipid metabolism [50]. Therefore, we hypothesized that dietary supplementation of broiler chickens with *L. paracasei* LK01 would help to enhance lipid metabolism, promote bone growth and metabolism, and thereby increase body weight.

Interleukins (IL-1β, IL-2, IL-6) are cytokines that leukocytes interact with during the immune response and have the role of transmitting information, activating and regulating immune cells [51]. TNF-α is an innate immune-associated cytokine with pro-inflammatory properties that are critical for host defense, induction of inflammation, and triggering of apoptosis [52]. In vitro studies have shown that *L. paracasei* can relieve lipopolysaccharide (LPS)-induced cell inflammation by reducing the expression of pro-inflammatory cytokines IL-1β, IL-2, and TNF-α and increasing the expression of the anti-inflammatory cytokine interleukin-10 (IL-10) [24,25]. In vivo studies have found that supplementing mice with *L. paracasei* PS23 significantly increases serum IL-10 levels and exhibits lower serum corticosterone levels [53]. In addition, Kang et al. [54] found that supplementing the diet of broilers with 10^7^ CFU/g of *L. paracasei* XLK401 significantly reduced the level of the pro-inflammatory factor IL-6 in the serum. Xiao et al. [35] found that drinking water supplemented with 2 × 10^8^ CFU/L *Lactobacillus plantarum* HJZW08 significantly reduced serum IL-2, IL-1β, IL-6, and TNF-α levels in broiler chickens. Our results showed that IL-1β levels decreased significantly in all experimental groups except 10^10^ group; TNF-α levels decreased significantly in the 10^7^, 10^8^, 10^9^, and 10^10^ groups.

In addition, we found that serum immunoglobulin (IgA, IgM, and IgG) levels were higher in the experimental group than in the control group, but the differences in IgA and IgG were not significant, whereas serum IgM levels were significantly higher in the 10^6^ and 10^7^ groups. The results of Zhang et al. [2] in *Bacillus coagulans* are almost the same as ours; his study found that feeding Bacillus coagulans to broiler chickens significantly reduced serum pro-inflammatory factors (IL-1β, IL-6, and TNF-α) levels, increased anti-inflammatory factor (IL-10) concentrations, and significantly elevated serum immunoglobulin (IgA, IgM, and IgY) levels. In addition, several studies have shown that probiotics can promote the production of relevant immunoglobulins (IgG, IgA, and IgM), thus enhancing the immunity of chickens [55,56,57]. Therefore, we hypothesized that supplementation with *L. paracasei* LK01 could likewise reduce inflammation by decreasing inflammatory gene expression, or increasing the production of anti-inflammatory factors, as with other probiotics, and could improve the immune function of the body by stimulating B-lymphocytes to enhance the production of immunoglobulins (IgG, IgA, and IgM).

T-SOD scavenges free radicals and protects cells from damage; MDA is a marker of oxidative stress and reflects the degree of lipid peroxidation in the body [55,56,57,58]. Studies have shown that sustained chronic heat stress increases the levels of MDA in blood and tissues and increases the activity of antioxidant enzymes [41]. In contrast, Liu et al. [59] found that the addition of 5 × 10^8^ cfu/kg of *Bacillus subtilis* HC6 to broiler diets significantly increased serum levels of T-AOC and T-SOD. T-AOC represents the total antioxidant level composed of various antioxidant substances and antioxidant enzymes, etc., and the higher its value, the higher the antioxidant capacity of broilers. In addition, in a study of the antioxidant effect of *Lactobacillus plantarum* on broilers with necrotic enteritis, it was found that the MDA content of the serum in the experimental group was reduced and the T-SOD activity was increased [60]. In addition, Chen et al. found that adding aflatoxin to the feed increased the MDA content and decreased the T-SOD activity in the livers of broiler chickens, while adding *Lactobacillus salivarius* increased the activity of antioxidant enzymes and decreased the MDA content [61]. Our results showed that the MDA level of the experimental group was significantly lower than that of the control group; in addition, the T-AOC and T-SOD levels of the experimental group were higher than those of the control group, but the difference was not significant. This suggests that dietary supplementation with *L. paracasei* LK01 may be similarly characterized to enhance antioxidant function in broilers.

The intestinal villi are located in the finger-like projections of the small intestinal epithelium and lamina propria that bulge into the intestinal lumen, which not only have the role of nutrient absorption, but also the villus oscillation pushes pathogenic microorganisms out of the way and filters out the harmful factors effectively [62]. Therefore, maintaining the integrity of the small intestinal epithelium is crucial for nutrient digestion and absorption, in which villus height and crypt depth are key factors [63,64]. A previous study found that dietary supplementation of broiler chickens with *Bacillus coagulans* and *Bacillus licheniformis* significantly elevated duodenal, jejunal, and intestinal villus lengths and significantly decreased duodenal crypt depth [43]. Another study found that dietary supplementation of broiler chickens with *Bacillus subtilis* HC6 increased jejunal and ileal villus heights and increased ileal V/C values [59]. Song et al. [65] showed that dietary supplementation with a mixture of *Bacillus licheniformis*, *Bacillus subtilis*, and *Lactobacillus plantarum* increased jejunal villus height and improved partial intestinal barrier function in broilers. In addition, Gyawali et al. [20] found that dietary supplementation of broiler chickens with a novel *L. paracasei* capsule significantly elevated villus height in all intestinal sections and enhanced V/C values. These studies showed the positive effects of probiotics on the broiler intestine, namely, an increase in villus height and V/C value and a decrease in crypt depth. In our study, we found no significant changes in villus height in all intestinal segments of broilers, but an improvement in crypt depth in some experimental groups compared to the control group was observed. In addition, group 10^6^ significantly increased the V/C value of the jejunum and ileum. This means that *L. paracasei* LK01 added to the diet can have a positive effect on the digestion and absorption of nutrients by maintaining the morphological health of the intestines, which is reflected to some extent in the lower FCR during the 28–35 d period.

Lipase, amylase, and protease, respectively, break down triglycerides, starch, and proteins; they promote the digestion and absorption of nutrients such as lipids, carbohydrates, and amino acids [66,67]. Therefore, the magnitude of activity of digestive enzymes is also one of the key factors affecting the growth and development of the organism. A previous study found that supplementing broiler diets with *Lactobacillus johnsonii* BS15 significantly increased protease and lipase activity in the small intestine at 21 days and lipase activity in the ileum at 42 days [68]. Jin et al. [69] found that a diet containing a mixture of 12 lactic acid bacteria significantly increased the level of amylase in the small intestine of chickens, but did not affect the activity of proteases and lipases in the small intestine. In addition, Wang et al. [70] found that supplementing broiler diets with *Bacillus coagulans* significantly increased the activity of protease and amylase in the duodenum. In our study, group 10^6^ significantly increased the protease activity of the duodenum, jejunum, and ileum, while group 10^10^ significantly increased the protease activity of the duodenum and jejunum. This suggests that dietary supplementation with *L. paracasei* LK01 has the potential to promote protease secretion in the small intestine of broilers.

The cecum is the most microbiologically diverse region of the gastrointestinal tract and possesses a complex, diverse, and stable microbial community. And the gut microbiota is not only an important barrier against invasive substances, but also regulates symbiotic homeostasis and normal physiological processes [71]. Therefore, the balance of intestinal microorganisms is of great importance in the production of livestock and poultry, and it is directly related to their health and production performance [72]. Previous studies have found that supplementing broiler diets with *Bacillus subtilis* had no effect on the α diversity index of the cecal flora, but significantly increased the β diversity index of the cecal microbiota [59,73]. Zhang et al. [2] showed the same results on *Bacillus coagulans,* where no significant changes in Shannon and Simpson indices were found in the experimental group, but the β diversity in the scatter plots of the principal component analysis and principal coordinate analysis showed a significant separation between the experimental group and the control group. Another study showed that adding 500 ppm encapsulated *L. paracasei* microcapsules to the diet of broilers had no effect on the α diversity index of the cecal flora but changed the structure and aggregation of the cecal flora [20]. Our research results are similar to this.

The PLS-DA plot (Figure 2B) shows that the dietary supplement *L. paracasei* LK01 caused a significant change in the microbial profile. It is well known that some bacteria in *Firmicutes* help to promote the digestion of cellulose in food and participate in a variety of metabolic pathways in the gut, so they are extremely important for the health of the host [74]. In contrast, *Bacteroidetes* phylum is mainly responsible for catabolizing polysaccharides and dietary fibers in the intestinal tract to produce short-chain fatty acids (SCFAs) such as acetic acid, propionic acid, and butyric acid, which are an important source of energy for the intestinal epithelial cells, as well as positively affecting the maintenance of the intestinal barrier function and the regulation of the immune system [75]. In addition, a lower ratio of *Firmicutes* to *Bacteroidetes* (F/B), which is usually associated with a high-fiber diet, is considered a healthier state of the intestinal microbiota and is associated with a lower risk of metabolic diseases [76]. Our results showed that the experimental group increased the proportion of *Bacteroidetes* and significantly reduced the F/B value. In addition, 10^6^ group also increased the proportion of Cyanobacteria and Desulfobacterota (Figure 2C). This is similar to the results of Xu et al. [7], where the cecal flora of broilers fed *L. paracasei* was dominated by *Firmicutes* and *Bacteroidetes*, accounting for more than 85% of the total number of microorganisms. Thus, our results suggest that dietary supplementation with *L. paracasei* LK01 optimized the structure of the cecum flora. At the genus level, we found that *ClostridiaUCG-014* and *Alistipes* were the dominant bacteria (Figure 2D,E); in addition, the 10^6^ group significantly increased the abundance of beneficial bacteria such as *Ruminococcaceae*, *Lachnospiraceae*, and *Faecalibacterium*, compared to the CON group. Some Clostridia are very important in the gut, such as butyric acid-producing *Clostridium*, which can help maintain the health of intestinal epithelial cells, enhance intestinal barrier function, and regulate the immune system [77], whereas *Alistipes*, a major member of the *Rikenellaceae* family, is a bile-resistant organism capable of producing fibrinolysin, digesting gelatin, and fermenting carbohydrates to produce acetic acid, and is often regarded as a beneficial bacterium for the intestinal tract [78]. These results indicate that dietary supplementation with *L. paracasei* LK01 has the potential to promote the secretion of short-chain fatty acids, improve the richness of the cecal flora, increase the abundance of beneficial bacteria, and maintain intestinal health.

## 5. Conclusions

Dietary supplementation of *L. paracasei* has the potential to improve the growth performance of broilers; to improve serum biochemical immunity indexes and serum antioxidant capacity of broilers; and to improve intestinal morphology, optimize the structure of intestinal flora, and maintain intestinal health. Thus, *L. paracasei* LK01 has potential as a dietary supplement for broilers. In summary, the findings from this study indicate that 10^6^ CFU/kg of *L. paracasei* is the optimal supplementation in broiler diets to increase overall performance and health.

## Figures and Tables

**Table 1:** Composition and nutrient levels of basic diets for broilers from 1 to 21 days of age.

Diets	Proportions (%)	Nutrient Content	
Corn	51.05	ME ^2^, KJ/Kg	1260
Soybean meal	29.46	CP, %	24.60
Corn gluten meal (63.5% CP)	5.50	Total P, %	0.63
Soybean oil	4.01	Ca, %	0.95
Standard powder	3.00	Met, %	0.57
Corn DDGS	2.00	Lys, %	1.38
Talcum powder	1.57	EE, %	6.89
Premix ^1^	1.00	CF, %	2.79
Monocalcium phosphate	0.78	Ash, %	4.84
L-Lysine sulfate	0.75		
NaCl	0.31		
DL-Methionine	0.21		
Montmorillonite	0.20		
L-Threonine	0.16		
Total	100.00		

^1^ The premix provided the following for per kg of basal diet in 1 to 21 days of age: vitamin A 1200 IU, vitamin D_3_ 3000 IU, vitamin E 30 IU, vitamin B_2_ 9 mg, vitamin K_3_ 6 mg, vitamin B_12_ 0. 03 mg, vitamin B_6_ 3 mg, biotin 0.165 mg, folic acid 0.9 mg, choline 0.35 g, Cu 6 mg, Mn 30 mg, Fe 35.28 mg, Zn 30 mg; ^2^ ME was a calculated value, while the others were measured values.

**Table 2:** Composition and nutrient levels of basic diets for broilers from 22 to 42 days of age.

Diets	Proportions (%)	Nutrient Content	
Corn	57.45	ME ^2^, KJ/Kg	1370
Soybean meal	16.08	CP, %	19.20
Lard	7.42	Total P, %	0.57
Corn gluten meal (60% CP)	6.00	Ca, %	0.85
Standard powder	3.00	Met, %	0.55
Corn DDGS	2.50	Lys, %	1.27
Talcum powder	1.52	EE, %	10.25
Nucleicacid residue ^3^	1.50	CF, %	1.74
Premix ^1^	1.00	Ash, %	4.28
Feather duster	1.00		
L-Lysine sulfate	0.95		
Monocalcium phosphate	0.68		
NaCl	0.30		
DL-Methionine	0.22		
Montmorillonite	0.20		
L-Threonine	0.15		
Tryptophan (Trp)	0.03		
Total	100.00		

^1^ The premix provided the following per kg of basal diet at 22 to 52 days of age: vitamin A 10,000 IU, vitamin D_3_ 2500 IU, vitamin E 25 IU, vitamin B_2_ 7.5 mg, vitamin K_3_ 5 mg, vitamin B_12_ 0.025 mg, vitamin B_6_ 5 mg, biotin 0.1375 mg, folic acid 1.5 mg, choline 0.3 g, Cu 5 mg, Mn 25 mg, Fe 29.25 mg, Zn 25 mg; ^2^ ME was a calculated value, while the others were measured values; ^3^ nucleotide residue is the solid residue remaining after the fermentation of glutamic acid bacillus and a medium consisting of plant-derived ingredients such as sucrose, honey, starch or their hydrolysates, and ammonium salts to produce disodium 5’-inosinate and disodium 5’-guanylate.

**Table 3:** Effects of *L. paracasei* LK01 on growth performance in broilers.

Items	CON	T1	T2	T3	T4	T5	*p*-Value
ADFI, g/d							
0–7 d	14.96 ± 0.21	15.36 ± 0.57	15.34 ± 0.19	15.44 ± 0.20	15.20 ± 0.09	15.50 ± 0.44	0.343
7–14 d	32.13 ± 0.15	32.04 ± 0.31	32.39 ± 0.43	32.09 ± 0.35	32.30 ± 0.46	32.62 ± 0.67	0.404
14–21 d	51.11 ± 1.13	51.36 ± 1.62	52.06 ± 1.44	51.34 ± 1.60	51.47 ± 1.42	51.91 ± 0.80	0.101
21–28 d	73.74 ± 1.25	74.25 ± 0.71	74.25 ± 1.41	75.18 ± 0.16	72.69 ± 2.26	72.23 ± 1.24	0.826
28–35 d	90.85 ± 2.66	91.45 ± 2.74	93.03 ± 1.02	92.48 ± 1.89	91.27 ± 3.38	91.49 ± 2.74	0.821
35–42 d	105.75 ± 2.87	110.73 ± 1.08	110.05 ± 2.73	110.59 ± 2.94	107.38 ± 5.18	109.02 ± 4.07	0.347
0–21 d	32.73 ± 0.50	32.92 ± 0.83	33.26 ± 0.69	32.96 ± 0.72	32.99 ± 0.66	33.34 ± 0.64	0.869
21–42 d	90.11 ± 2.26	92.14 ± 1.51	92.44 ± 1.72	92.75 ± 1.65	90.45 ± 3.61	90.91 ± 2.68	0.670
0–42 d	61.42 ± 1.38	62.53 ± 1.17	62.85 ± 1.21	62.86 ± 1.19	61.72 ± 2.14	62.13 ± 1.66	0.798
BW, g							
21 d	537 ± 31.25 ^c^	572 ± 21.90 ^a^	571 ± 26.13 ^a^	558 ± 28.08 ^ab^	551 ± 24.58 ^b^	563 ± 23.91 ^ab^	0.001
28 d	896 ± 32.77	931 ± 32.12	931 ± 36.65	909 ± 36.51	908 ± 34.78	899 ± 34.03	0.115
35 d	1236 ± 87.41 ^b^	1291 ± 84.29 ^a^	1296 ± 56.38 ^a^	1250 ± 85.26 ^ab^	1241 ± 84.27 ^b^	1269 ± 89.41 ^ab^	0.050
42 d	1526 ± 152.18	1685 ± 117.81	1655 ± 146.38	1612 ± 146.13	1530 ± 182.68	1602 ± 166.18	0.357
ADG, g/d							
21–28 d	51.01 ± 4.74	51.39 ± 5.73	51.21 ± 5.34	52.69 ± 5.25	51.06 ± 5.81	51.06 ± 5.81	0.814
28–35 d	50.00 ± 6.42	50.31 ± 5.74	52.14 ± 5.53	50.40 ± 5.86	50.27 ± 5.34	50.90 ± 6.18	0.422
35–42 d	50.46 ± 6.70	54.56 ± 4.98	51.59 ± 4.69	52.50 ± 5.22	50.68 ± 4.92	51.77 ± 2.87	0.334
0–21 d	23.81 ± 1.49	25.48 ± 1.04	25.43 ± 1.24	24.81 ± 1.34	24.48 ± 1.17	25.05 ± 1.14	0.580
21–42 d	47.10 ± 5.76	53.00 ± 4.57	51.62 ± 5.73	50.19 ± 5.62	46.62 ± 7.53	49.48 ± 6.77	0.761
0–42	35.46 ± 3.63	39.24 ± 2.81	38.53 ± 3.49	37.5 ± 3.48	35.55 ± 4.35	37.27 ± 3.96	0.755
FCR							
21–28 d	1.48 ± 0.21	1.47 ± 0.18	1.43 ± 0.19	1.46 ± 0.19	1.48 ± 0.22	1.43 ± 0.21	0.683
28–35 d	2.00 ± 0.35 ^a^	1.83 ± 0.30 ^bc^	1.78 ± 0.22 ^c^	1.81 ± 0.30 ^bc^	1.98 ± 0.42 ^ab^	1.89 ± 0.33 ^abc^	0.021
35–42 d	2.17 ± 0.37	1.97 ± 0.28	2.01 ± 0.29	2.06 ± 0.39	2.04 ± 0.26	2.04 ± 0.37	0.255
0–21 d	1.37 ± 0.34	1.29 ± 0.80	1.32 ± 0.56	1.33 ± 0.54	1.35 ± 0.56	1.33 ± 0.56	0.645
21–42 d	1.91 ± 0.39	1.76 ± 0.33	1.79 ± 0.30	1.85 ± 0.29	1.94 ± 0.49	1.84 ± 0.40	0.153
0–42 d	1.73 ± 0.38	1.62 ± 0.42	1.63 ± 0.35	1.68 ± 0.34	1.72 ± 0.49	1.68 ± 0.56	0.329

One-way ANOVA revealed treatment main effects. CON: control group, basal diet; T1: basal diet + 10^6^ CFU/kg *L. paracasei* LK01; T2: basal diet + 10^7^ CFU/kg *L. paracasei* LK01; T3: basal diet + 10^8^ CFU/kg *L. paracasei* LK 01; T4: basal diet + 10^9^ CFU/kg *L. paracasei* LK01; T5: basal diet + 10^10^ CFU/kg *L. paracasei* LK01. ^a,b,c^ Means within a row with different letters are statistically significant (*p* < 0.05). The experimental results are expressed as the mean ± standard error of the mean (SEM).

**Table 4:** Effects of *L. paracasei* LK01 on serum physiological and biochemical indexes of broilers.

Items	CON	T1	T2	T3	T4	T5	*p*-Value
ALT, U/L	30.49 ± 3.24 ^a^	30.31 ± 2.62 ^a^	30.78 ± 2.77 ^a^	23.24 ± 0.90 ^b^	25.96 ± 3.06 ^ab^	29.07 ± 2.02 ^a^	0.050
AST, U/L	35.23 ± 3.93 ^a^	25.28 ± 1.54 ^b^	30.08 ± 3.19 ^ab^	25.54 ± 3.15 ^b^	30.06 ± 5.16 ^ab^	24.13 ± 4.26 ^b^	0.012
ALP, ng/ml	8.02 ± 0.64	9.42 ± 0.26	8.97 ± 0.74	8.58 ± 0.23	8.68 ± 0.32	8.24 ± 1.14	0.166
TP, mg/mL	80.92 ± 2.85	83.89 ± 3.76	86.02 ± 1.19	81.85 ± 1.57	83.69 ± 1.92	82.92 ± 2.49	0.083
ALB, mg/mL	42.52 ± 0.93	43.42 ± 2.04	42.52 ± 0.64	42.07 ± 1.59	42.28 ± 0.77	42.58 ± 1.09	0.672
UA, mmol/L	367.5 ± 22.83 ^a^	348.7 ± 24.10 ^a^	252.1 ± 22.46 ^b^	268.6 ± 15.79 ^b^	255.2 ± 23.17 ^b^	294.1 ± 27.87 ^ab^	0.003
TC, mmol/L	4.66 ± 0.35 ^a^	4.51 ± 0.19 ^a^	3.81± 0.30 ^b^	3.83± 0.21 ^b^	3.62± 0.27 ^b^	4.64 ± 0.37 ^a^	0.007
TG, mmol/L	5.80 ± 0.26 ^a^	4.54 ± 0.32 ^c^	4.87 ± 0.33 ^bc^	5.44 ± 0.35 ^ab^	5.35 ± 0.25 ^ab^	5.43 ± 0.26 ^ab^	<0.001
P, mmol/L	10.38 ± 1.34	11.37 ± 1.63	11.63 ± 1.41	10.77 ± 1.51	10.59 ± 1.23	11.66 ± 0.75	0.374
Ca, mmol/L	4.96 ± 0.35	4.51 ± 0.52	4.81 ± 0.36	5.16 ± 0.26	4.52 ± 0.22	4.56 ± 0.38	0.186

One-way ANOVA revealed treatment main effects. CON: control group, basal diet; T1: basal diet + 10^6^ CFU/kg *L. paracasei* LK01; T2: basal diet + 10^7^ CFU/kg *L. paracasei* LK01; T3: basal diet + 10^8^ CFU/kg *L. paracasei* LK 01; T4: basal diet + 10^9^ CFU/kg *L. paracasei* LK01; T5: basal diet + 10^10^ CFU/kg *L. paracasei* LK01. ^a,b,c^ Means within a row with different letters are statistically significant (*p* < 0.05). The experimental results are expressed as the mean ± standard error of the mean (SEM).

**Table 5:** Effect of *L. paracasei* LK01 on serum immune and antioxidant indices in broilers.

Items	CON	T1	T2	T3	T4	T5	*p*-Value
IgA (ng/mL)	440.27 ± 10.89	470.96 ± 7.65	454.68 ± 15.53	448.61 ± 9.93	457.14 ± 14.11	457.48 ± 24.87	0.124
IgG (μg/mL)	12.52 ± 0.16	13.93± 0.90	13.76 ± 0.08	12.44± 0.10	13.42 ± 0.74	13.23 ± 0.55	0.072
IgM (ng/mL)	355.64 ± 13.61 ^b^	393.10 ± 11.06 ^a^	387.53 ± 16.07 ^a^	371.84 ± 9.25 ^ab^	360.30 ± 6.92 ^b^	370.04 ± 11.44 ^ab^	0.024
T-SOD (U/mL)	98.37 ± 1.17	103.95 ± 3.29	108.26 ± 3.36	102.3 ± 3.74	103.64 ± 0.48	104.32 ± 3.03	0.088
GSH-Px (mIU/mL)	25.51 ± 1.47	26.82 ± 1.62	25.96 ± 1.55	25.78 ± 1.27	25.58 ± 0.93	26.13 ± 2.62	0.935
T-AOC (U/L)	108.24 ± 5.77	110.81 ± 4.94	119.98 ± 6.05	110.4 ± 9.67	119.98 ± 3.48	117.26 ± 4.88	0.065
MDA (pmol/L)	5.67 ± 0.47 ^a^	4.18 ± 0.21 ^b^	3.91 ± 0.60 ^b^	4.4 ± 0.37 ^b^	3.94 ± 0.46 ^b^	4.51 ± 0.18 ^b^	<0.001
IL-1 β (pg/mL)	177.83 ± 0.42 ^a^	154.11 ± 2.19 ^b^	154.75 ± 6.48 ^b^	154.51 ± 6.96 ^b^	157.77 ± 6.53 ^b^	170.15 ± 8.84 ^a^	<0.001
IL-2 (pg/mL)	199.34 ± 7.59	200.94 ± 9.46	204.97 ± 4.96	204.40 ± 9.42	201.86 ± 5.22	204.47 ± 4.59	0.857
IL-6 (pg/mL)	156.30 ± 9.10	129.41 ± 16.56	128.99 ± 6.96	137.22 ± 12.65	135.06 ± 5.16	130.97 ± 13.02	0.092
TNF-α (pg/mL)	199.07 ± 5.98 ^a^	188.22 ± 2.59 ^ab^	185.49 ± 6.37 ^b^	179.57 ± 13.33 ^b^	182.15 ± 7.02 ^b^	183.83 ± 13.51 ^b^	0.056

One-way ANOVA revealed treatment main effects. CON: control group, basal diet; T1: basal diet + 10^6^ CFU/kg *L. paracasei* LK01; T2: basal diet + 10^7^ CFU/kg *L. paracasei* LK01; T3: basal diet + 10^8^ CFU/kg *L. paracasei* LK 01; T4: basal diet + 10^9^ CFU/kg *L. paracasei* LK01; T5: basal diet + 10^10^ CFU/kg *L. paracasei* LK01. ^a,b^ Means within a row with different letters are statistically significant (*p* < 0.05). The experimental results are expressed as the mean ± standard error of the mean (SEM).

**Table 6:** Effect of *L. paracasei* LK01 on the intestinal morphology of broilers.

Items	CON	T1	T2	T3	T4	T5	*p*-Value
Duodenum							
Villus height, μm	1489.51 ± 110.27	1556.34 ± 79.18	1412.43 ± 60.64	1427.00 ± 40.85	1548.87 ± 77.77	1468.10 ± 126.40	0.156
Crypt depth, μm	98.59 ± 2.27 ^a^	91.34 ± 5.70 ^ab^	96.01 ± 6.29 ^ab^	87.13 ± 1.73 ^b^	89.84 ± 11.54 ^ab^	91.49 ± 6.78 ^ab^	0.034
V/C	15.58 ± 0.81	16.20 ± 0.94	18.61 ± 0.76	17.51 ± 0.70	17.33 ± 1.88	15.94 ± 0.58	0.104
Jejunum							
Villus height, μm	1349.46 ± 93.74	1476.86 ± 79.47	1461.54 ± 81.50	1321.07 ± 91.43	1424.35 ± 189.85	1364.14 ± 151.82	0.307
Crypt depth, μm	94.37 ± 0.40	87.62 ± 10.44	87.52 ± 8.58	90.66 ± 3.52	88.81 ± 2.41	88.97 ± 7.64	0.832
V/C	13.42 ± 0.90 ^b^	15.31 ± 0.19 ^a^	13.51 ± 0.13 ^b^	13.25 ± 0.09 ^b^	14.92 ± 0.98 ^ab^	14.34 ± 0.77 ^ab^	0.050
Ileum							
Villus height, μm	975.63 ± 79.51	914.33 ± 5.12	978.86 ± 96.59	897.86 ± 96.85	1015.70 ± 6.30	1009.00 ± 80.44	0.301
Crypt depth, μm	99.50 ± 1.37 ^a^	83.54 ± 4.29 ^b^	84.87 ± 0.13 ^b^	91.81 ± 6.18 ^ab^	87.23 ± 4.32 ^b^	93.57 ± 2.91 ^ab^	0.044
V/C	9.13 ± 1.04 ^b^	11.14 ± 0.76 ^a^	9.30 ± 0.35 ^b^	10.40 ± 0.06 ^ab^	9.41 ± 0.22 ^b^	9.98 ± 0.66 ^ab^	0.031

One-way ANOVA revealed treatment main effects. CON: control group, basal diet; T1: basal diet + 10^6^ CFU/kg *L. paracasei* LK01; T2: basal diet + 10^7^ CFU/kg *L. paracasei* LK01; T3: basal diet + 10^8^ CFU/kg *L. paracasei* LK 01; T4: basal diet + 10^9^ CFU/kg *L. paracasei* LK01; T5: basal diet + 1010 CFU/kg *L. paracasei* LK01. ^a,b^ Means within a row with different letters are statistically significant (*p* < 0.05). The experimental results are expressed as the mean ± standard error of the mean (SEM).

**Table 7:** Effect of *L. paracasei* LK01 on the activity of intestinal digestive enzymes in broilers.

Items	CON	T1	T2	T3	T4	T5	*p*-Value
Duodenum							
Protease, U/mL	1084.85 ± 95.81 ^b^	1451.72 ± 81.53 ^a^	1276.87 ± 45.65 ^ab^	1151.34 ± 140.43 ^b^	1239.66 ± 60.99 ^ab^	1384.55 ± 183.11 ^a^	0.014
Amylase, IU/L	276.18 ± 18.78	291.15 ± 46.64	329.37 ± 21.56	311.85 ± 32.24	342.65 ± 4.54	299.02 ± 48.03	0.157
Lipase, U/L	731.61 ± 41.73	733.32 ± 40.94	791.83 ± 40.49	765.75 ± 55.43	729.93 ± 28.13	758.50 ± 14.19	0.261
Jejunum							
Protease, U/mL	1031.65 ± 58.84 ^b^	1379.09 ± 42.70 ^a^	1124.67 ± 35.83 ^b^	1158.51 ± 45.65 ^b^	1026.70 ± 57.06 ^b^	1361.50 ± 100.71 ^a^	0.003
Amylase, IU/L	257.85 ± 46.91	306.15 ± 26.11	292.14 ± 46.86	289.35 ± 47.69	305.42 ± 52.93	262.16 ± 39.69	0.536
Lipase, U/L	770.03 ± 35.69	770.97 ± 36.12	835.70 ± 40.24	814.21 ± 50.73	784.47 ± 21.97	810.35 ± 82.08	0.272
Ileum							
Protease, U/mL	1111.89 ± 61.34 ^b^	1408.68 ± 49.46 ^a^	1252.66 ± 102.71 ^ab^	1147.75 ± 63.43 ^b^	1166.62 ± 66.25 ^b^	1193.03 ± 152.53 ^b^	0.047
Amylase, IU/L	267.17 ± 35.81	280.17 ± 46.13	309.75 ± 26.06	284.55 ± 67.33	320.25 ± 32.67	291.41 ± 66.61	0.615
Lipase, U/L	770.41 ± 27.36	776.56 ± 86.34	802.00 ± 67.59	774.02 ± 56.77	796.15 ± 65.22	786.10 ± 14.19	0.963

One-way ANOVA revealed treatment main effects. CON: control group, basal diet; T1: basal diet + 10^6^ CFU/kg *L. paracasei* LK01; T2: basal diet + 10^7^ CFU/kg *L. paracasei* LK01; T3: basal diet + 10^8^ CFU/kg *L. paracasei* LK 01; T4: basal diet + 10^9^ CFU/kg *L. paracasei* LK01; T5: basal diet + 10^10^ CFU/kg *Lactobacillus paracasei* LK01. ^a,b^ Means within a row with different letters are statistically significant (*p* < 0.05). The experimental results are expressed as the mean ± standard error of the mean (SEM).

## Data Availability

The 16S rRNA sequencing data for all the samples were deposited into the NCBI Se-quence Read Archive (SRA) under accession number PRJNA1174407 (https://www.ncbi.nlm.nih.gov/bioproject/PRJNA1174407, accessed on 18 October 2024).

## Appendix A. Information on All the Kits Used to Measure Serum Biochemicals and Intestinal Digestive Enzymes in Broilers

IgA	IgA ELISA Kit	A045-4-1
IgM	IgM ELISA Kit	A110-1-1
IgG	IgG ELISA Kit	A110-1-1
T-SOD	Superoxide Dismutase (SOD) Assay Kit	A00-3-2
GSH/Px	Glutathione Peroxidase (GSH-Px) Kit	A005-1-1
T-AOC	Total Antioxidant Capacity (T-AOC) Kit	A015-2-1
MDA	Malondialdehyde (MDA) Kit	H003-1-2
IL-1β	Interleukin-1β Assay Kit	H002-1-2
IL-2	Interleukin-2 Assay Kit	H003-1-1
IL-6	Interleukin-6 Assay Kit	H007-1-1
Protease (U/mL)	Protease assay kit	W060-1-1
Lipase (U/L)	Lipase (LPS) assay kit	W054-1-1
Amylase(U/dL)	Total amylase (T-AMY) assay kit	C016-1-1

## References

[1] Mottet A., Tempio G. (2017). Global poultry production: Current state and future outlook and challenges. World’s Poult. Sci. J..

[2] Zhang B., Zhang H., Yu Y., Zhang R., Wu Y., Yue M., Yang C. (2021). Effects of *Bacillus coagulans* on growth performance, antioxidant capacity, immunity function, and gut health in broilers. Poult. Sci..

[3] Mehdi Y., Letourneau-Montminy M.P., Gaucher M.L., Chorfi Y., Suresh G., Rouissi T., Brar S.K., Cote C., Ramirez A.A., Godbout S. (2018). Use of antibiotics in broiler production: Global impacts and alternatives. Anim. Nutr..

[4] Neveling D.P., Dicks L.M.T. (2021). Probiotics: An antibiotic replacement strategy for healthy broilers and productive rearing. Probiotics Antimicrob. Proteins.

[5] Abd El-Hack M.E., El-Saadony M.T., Salem H.M., El-Tahan A.M., Soliman M.M., Youssef G.B.A., Taha A.E., Soliman S.M., Ahmed A.E., El-kott A.F. (2022). Alternatives to antibiotics for organic poultry production: Types, modes of action and impacts on bird’s health and production. Poult. Sci..

[6] Huang C., Li S., Huang L., Watanabe K. (2018). Identification and Classification for the *Lactobacillus casei* Group. Front. Microbiol..

[7] Xu Y., Tian Y., Cao Y., Li J., Guo H., Su Y., Tian Y., Wang C., Wang T., Zhang L. (2019). Probiotic properties of *Lactobacillus paracasei* subsp. *paracasei* L1 and its growth performance-promotion in chicken by improving the intestinal microflora. Front. Physiol..

[8] Sornsenee P., Singkhamanan K., Sangkhathat S., Saengsuwan P., Romyasamit C. (2021). Probiotic properties of *Lactobacillus species* isolated from fermented palm sap in Thailand. Probiotics Antimicrob. Proteins.

[9] Liu N., Miao S., Qin L. (2020). Screening and application of lactic acid bacteria and yeasts with l-lactic acid-producing and antioxidant capacity in traditional fermented rice acid. Food Sci. Nutr..

[10] Zhang S., Han B., Mao Y., Zhang Z., Li Z., Kong C., Wu Y., Chen G., Wang L. (2022). *Lacticaseibacillus paracasei SH2020* induced antitumor immunity and synergized with anti-programmed cell death 1 to reduce tumor burden in mice. Gut Microbes.

[11] El-Saadony M.T., Saad A.M., Taha T.F., Najjar A.A., Zabermawi N.M., Nader M.M., AbuQamar S.F., El-Tarabily K.A., Salama A. (2021). Selenium nanoparticles, from *Lactobacillus paracasei* HM1 capable of antagonizing animal pathogenic fungi, as a new source from human breast milk. Saudi J. Biol. Sci..

[12] Gudiña E.J., Rocha V., Teixeira J.A., Rodrigues L.R. (2010). Antimicrobial and antiadhesive properties of a biosurfactant isolated from *Lactobacillus paracasei* ssp. *paracasei* A_20_. Lett. Appl. Microbiol..

[13] Cheng L., Chou P., Hou A., Huang C., Shiu W., Wang S. (2022). *Lactobacillus paracasei* PS_23_ improves cognitive deficits via modulating the hippocampal gene expression and the gut microbiota in D-galactose- induced aging mice. Food Funct..

[14] Suo H., Liu S., Li J., Ding Y., Wang H., Zhang Y., Zhao X., Song J. (2018). *Lactobacillus paracasei* ssp. paracasei YBJ01 reduced d-galactose- induced oxidation in male Kuming mice. J. Dairy Sci..

[15] Da Silva Duarte V., Dos Santos Cruz B.C., Tarrah A., Sousa Dias R., de Paula Dias Moreira L., Lemos Junior W.J.F., Fidelis Silva L.C., Rocha Santana G., Licursi De Oliveira L., Gouveia Peluzio M.D.C. (2020). Chemoprevention of DMH-Induced Early Colon Carcinogenesis in Male BALB/c Mice by Administration of *Lactobacillus Paracasei* DTA81. Microorganisms.

[16] Shankar T., Palpperumal S., Kathiresan D., Sankaralingam S., Balachandran C., Baskar K., Hashem A., Alqarawi A.A., Abd Allah E.F. (2021). Biomedical and therapeutic potential of exopolysaccharides by *Lactobacillus paracasei* isolated from sauerkraut: Screening and characterization. Saudi J. Biol. Sci..

[17] Belviso S., Giordano M., Dolci P., Zeppa G. (2009). In vitro cholesterol-lowering activity of *Lactobacillus plantarum* and *Lactobacillus paracasei* strains isolated from the Italian Castelmagno PDO cheese. Dairy Sci. Technol..

[18] Tarrah A., Dos Santos Cruz B.C., Sousa Dias R., Silva Duarte V., Pakroo S., Licursi De Oliveira L., Gouveia Peluzio M.C., Corich V., Giacomini A., Oliveira De Paula S. (2021). *Lactobacillus paracasei* DTA81, a cholesterol-lowering strain having immunomodulatory activity, reveals gut microbiota regulation capability in balb/c mice receiving high-fat diet. J. Appl. Microbiol..

[19] Yang L., Xie X., Li Y., Wu L., Fan C., Liang T., Xi Y., Yang S., Li H., Zhang J. (2021). Evaluation of the Cholesterol-Lowering Mechanism of *Enterococcus faecium* Strain 132 and *Lactobacillus paracasei* Strain 201 in Hypercholesterolemia Rats. Nutrients.

[20] Gyawali I., Zeng Y., Zhou J., Li J., Wu T., Shu G., Jiang Q., Zhu C. (2022). Effect of novel *Lactobacillus paracaesi* microcapsule on growth performance, gut health and microbiome community of broiler chickens. Poult. Sci..

[21] Kim W., Jang Y.J., Han D.H., Jeon K., Lee C., Han H.S., Ko G. (2020). *Lactobacillus paracasei* KBL_382_ administration attenuates atopic dermatitis by modulating immune response and gut microbiota. Gut Microbes.

[22] Wang W., Li Q., Chai W., Sun C., Zhang T., Zhao C., Yuan Y., Wang X., Liu H., Ye H. (2019). *Lactobacillus paracasei* Jlus66 extenuate oxidative stress and inflammation via regulation of intestinal flora in rats with non alcoholic fatty liver disease. Crit. Rev. Food Sci. Nutr..

[23] Song E., Lee E., Kim Y.I., Shin D., Eom J., Shin H.S., Lee S., Nam Y. (2023). Gut microbial change after administration of *Lacticaseibacillus paracasei* AO_356_ is associated with anti-obesity in a mouse model. Front. Endocrinol..

[24] Choi J.H., Moon C.M., Shin T., Kim E.K., McDowell A., Jo M., Joo Y.H., Kim S., Jung H., Shim K. (2020). *Lactobacillus paracasei*-derived extracellular vesicles attenuate the intestinal inflammatory response by augmenting the endoplasmic reticulum stress pathway. Exp. Mol. Med..

[25] Suzuki H., Yamazaki T., Ohshio K., Sugamata M., Yoshikawa M., Kanauchi O., Morita Y. (2020). A specific strain of lactic acid bacteria, *Lactobacillus Paracasei*, inhibits inflammasome activation in vitro and prevents inflammation-related disorders. J. Immunol..

[26] Mo S.J., Nam B., Bae C., Park S., Shim J., Lee J. (2021). Characterization of novel *Lactobacillus paracasei* HY7017 capable of improving physiological properties and immune enhancing effects using red ginseng extract. Fermentation..

[27] Gyawali I., Zhou G., Xu G., Zeng Y., Li J., Zhou J., Jiang Q., Shu G., Zhu C. (2021). Oral Delivery of Lactobacillus paracasei via Microcapsule Modulates Gut Health and Intestinal Microbiota.

[28] Arai S., Iwabuchi N., Takahashi S., Xiao J., Abe F., Hachimura S. (2018). Orally administered heat-killed *Lactobacillus paracasei* MCC_1849_ enhances antigen-specific iga secretion and induces follicular helper t cells in mice. PLoS ONE.

[29] Gao J., Li Q., Liu Y., Yang B., Ahmed Sadiqb F., Li X., Mi S., Sang Y. (2022). Immunoregulatory effect of *Lactobacillus Paracasei* Vl8 exopolysaccharide on RAW264.7 cells by NF-kB and MAPK pathways. J. Funct. Foods.

[30] Liu H., Zhang J., Zhang S., Yang F., Thacker P.A., Zhang G., Qiao S., Ma X. (2014). Oral administration of *Lactobacillus fermentum* I5007 favors intestinal development and alters the intestinal microbiota in formula-fed piglets. J. Agric. Food Chem..

[31] Chen S., Zhou Y., Chen Y., Gu J. (2018). Fastp: An ultra-fast all-in-one FASTQ preprocessor. Bioinformatics.

[32] Magoc T., Salzberg S.L. (2011). Flash: Fast length adjustment of short reads to improve genome assemblies. Bioinformatics.

[33] Edgar R.C. (2013). Uparse: Highly accurate otu sequences from microbial amplicon reads. Nat. Methods.

[34] Stackebrandt E., Goebel B.M. (1994). Taxonomic note: A place for DNA-DNA reassociation and 16S rRNA sequence analysis in the present species definition in bacteriology. Int. J. Syst. Evol. Microbiol..

[35] Xiao X., Cui T., Qin S., Wang T., Liu J., Sa L., Wu Y., Zhong Y., Yang C. (2024). Beneficial effects of Lactobacillus Plantarum on growth performance, immune status, antioxidant function and intestinal microbiota in broilers. Poult. Sci..

[36] Leal K., Truong L., Maga E., King A. (2023). *Lactobacillus* (L. Plantarum & L. Rhamnosus) and Saccharomyces (s. Cerevisiae): Effects on performance, biochemical parameters, ammonium ion in manure, and digestibility of broiler chickens. Poult. Sci..

[37] Kalavathy R., Abdullah N., Jalaludin S., Ho Y.W. (2003). Effects of *Lactobacillus cultures* on growth performance, abdominal fat deposition, serum lipids and weight of organs of broiler chickens. Br. Poult. Sci..

[38] Peng Q., Zeng X.F., Zhu J.L., Wang S., Liu X.T., Hou C.L., Thacker P.A., Qiao S.Y. (2016). Effects of dietary *Lactobacillus plantarum* B_1_ on growth performance, intestinal microbiota, and short chain fatty acid profiles in broiler chickens. Poult. Sci..

[39] Bai S.P., Wu A.M., Ding X.M., Lei Y., Bai J., Zhang K.Y., Chio J.S. (2013). Effects of probiotic-supplemented diets on growth performance and intestinal immune characteristics of broiler chickens. Poult. Sci..

[40] Rashidi N., Khatibjoo A., Taherpour K., Akbari-Gharaei M., Shirzadi H. (2020). Effects of licorice extract, probiotic, toxin binder and poultry litter biochar on performance, immune function, blood indices and liver histopathology of broilers exposed to aflatoxin-B_1_. Poult. Sci..

[41] Yilmaz E., Gul M. (2023). Correction to: Effects of cumin (*Cuminum cyminum* L.) Essential oil and chronic heat stress on growth performance, carcass characteristics, serum biochemistry, antioxidant enzyme activity, and intestinal microbiology in broiler chickens. Vet. Res. Commun..

[42] Haque I., Ahmad N., Miah M.A. (2017). Comparative analysis of body weight and serum biochemistry in broilers supplemented with some selected probiotics and antibiotic growth promoters. J. Adv. Vet. Anim. Res..

[43] Elleithy E.M.M., Bawish B.M., Kamel S., Ismael E., Bashir D.W., Hamza D., Fahmy K.N.E. (2023). Influence of dietary *Bacillus Coagulans* and/or *Bacillus licheniformis*-based probiotics on performance, gut health, gene expression, and litter quality of broiler chickens. Trop. Anim. Health Prod..

[44] Nwaigwe C.U., Ihedioha J.I., Shoyinka S.V., Nwaigwe C.O. (2020). Evaluation of the hematological and clinical biochemical markers of stress in broiler chickens. Vet. World.

[45] Zhan H.Q., Dong X.Y., Li L.L., Zheng Y.X., Gong Y.J., Zou X.T. (2019). Effects of dietary supplementation with *Clostridium butyricum* on laying performance, egg quality, serum parameters, and cecal microflora of laying hens in the late phase of production. Poult. Sci..

[46] Salehizadeh M., Modarressi M.H., Mousavi S.N., Ebrahimi M.T. (2019). Effects of probiotic lactic acid bacteria on growth performance, carcass characteristics, hematological indices, humoral immunity, and IGF-I gene expression in broiler chicken. Trop. Anim. Health Prod..

[47] Shokryazdan P., Faseleh Jahromi M., Liang J.B., Ramasamy K., Sieo C.C., Ho Y.W. (2017). Effects of a *Lactobacillus salivarius* mixture on performance, intestinal health and serum lipids of broiler chickens. PLoS ONE.

[48] Ahmat M., Cheng J., Abbas Z., Cheng Q., Fan Z., Ahmad B., Hou M., Osman G., Guo H., Wang J. (2021). Effects of *Bacillus amyloliquefaciens* LFB_112_ on growth performance, carcass traits, immune, and serum biochemical response in broiler chickens. Antibiotics.

[49] Shannon T.A., Ledoux D.R., Rottinghaus G.E., Shaw D.P., Dakovic A., Markovic M. (2017). The efficacy of raw and concentrated bentonite clay in reducing the toxic effects of aflatoxin in broiler chicks. Poult. Sci..

[50] Zhang Q.Q., Chang C., Chu Q., Wang H.H., Zhang J., Yan Z.X., Song Z.G., Geng A.L. (2023). Dietary calcium and non-phytate phosphorus levels affect the performance, serum biochemical indices, and lipid metabolism in growing pullets. Poult. Sci..

[51] Rajput I.R., Li L.Y., Xin X., Wu B.B., Juan Z.L., Cui Z.W., Yu D.Y., Li W.F. (2013). Effect of *Saccharomyces boulardii* and *Bacillus subtilis* B_10_ on intestinal ultrastructure modulation and mucosal immunity development mechanism in broiler chickens. Poult. Sci..

[52] Al-Banna N.A., Cyprian F., Albert M.J. (2018). Cytokine responses in campylobacteriosis: Linking pathogenesis to immunity. Cytokine Growth Factor Rev..

[53] Liao J.F., Hsu C.C., Chou G.T., Hsu J.S., Liong M.T., Tsai Y.C. (2019). *Lactobacillus paracasei* PS_23_ reduced early-life stress abnormalities in maternal separation mouse model. Benef. Microbes.

[54] Kang X., Li X., Zhou H., Wang F., Lin L. (2023). Genome-wide and 16s rRNA sequencing-based analysis on the health effects of *Lacticaseibacillus paracasei* XLK_401_ on chicks. Microorganisms.

[55] Wang J., Yao L., Su J., Fan R., Zheng J., Han Y. (2023). Effects of Lactobacillus Plantarum and its fermentation products on growth performance, immune function, intestinal ph, and cecal microorganisms of lingnan yellow chicken. Poult. Sci..

[56] Song X., Lin Z., Yu C., Qiu M., Peng H., Jiang X., Du H., Li Q., Liu Y., Zhang Z. (2022). Effects of *Lactobacillus plantarum* on growth traits, slaughter performance, serum markers and intestinal bacterial community of Daheng broilers. J. Anim. Physiol. Anim. Nutr..

[57] Wu X.Z., Wen Z.G., Hua J.L. (2019). Effects of dietary inclusion of *Lactobacillus* and inulin on growth performance, gut microbiota, nutrient utilization, and immune parameters in broilers. Poult. Sci..

[58] Birben E., Sahiner U.M., Sackesen C., Erzurum S., Kalayci O. (2012). Oxidative stress and antioxidant defense. World Allergy Organ. J..

[59] Liu S., Xiao G., Wang Q., Zhang Q., Tian J., Li W., Gong L. (2023). Effects of dietary *Bacillus subtilis* HC6 on growth performance, antioxidant capacity, immunity, and intestinal health in broilers. Animals.

[60] Cao L., Wu X.H., Bai Y.L., Wu X.Y., Gu S.B. (2019). Anti-inflammatory and antioxidant activities of probiotic powder containing *Lactobacillus plantarum* 1.2567 in necrotic enteritis model of broiler chickens. Livest. Sci..

[61] Chen X., Ishfaq M., Wang J. (2022). Effects of *Lactobacillus salivarius* supplementation on the growth performance, liver function, meat quality, immune responses and salmonella pullorum infection resistance of broilers challenged with aflatoxin B_1_. Poult. Sci..

[62] Liu S., Xiao G., Wang Q., Tian J., Feng X., Zhang Q., Gong L. (2023). Effects of dietary astragalus membranaceus and codonopsis pilosula extracts on growth performance, antioxidant capacity, immune status, and intestinal health in broilers. Front. Vet. Sci..

[63] Xu Z.R., Hu C.H., Xia M.S., Zhan X.A., Wang M.Q. (2003). Effects of dietary fructooligosaccharide on digestive enzyme activities, intestinal microflora and morphology of male broilers. Poult. Sci..

[64] Samanya M., Yamauchi K. (2002). Histological alterations of intestinal villi in chickens fed dried *Bacillus subtilis* var. Natto. Comp. Biochem. Physiol. Part A Mol. Integr. Physiol..

[65] Song J., Xiao K., Ke Y.L., Jiao L.F., Hu C.H., Diao Q.Y., Shi B., Zou X.T. (2014). Effect of a probiotic mixture on intestinal microflora, morphology, and barrier integrity of broilers subjected to heat stress. Poult. Sci..

[66] Noy Y., Sklan D. (1995). Digestion and absorption in the young chick. Poult. Sci..

[67] Al-Marzooqi W., Leeson S. (2000). Effect of dietary lipase enzyme on gut morphology, gastric motility, and long-term performance of broiler chicks. Poult. Sci..

[68] Wang H., Ni X., Qing X., Zeng D., Luo M., Liu L., Li G., Pan K., Jing B. (2017). Live probiotic *Lactobacillus johnsonii* BS_15_ promotes growth performance and lowers fat deposition by improving lipid metabolism, intestinal development, and gut microflora in broilers. Front. Microbiol..

[69] Jin L.Z., Ho Y.W., Abdullah N., Jalaludin S. (2000). Digestive and bacterial enzyme activities in broilers fed diets supplemented with Lactobacillus cultures. Poult. Sci..

[70] Wang Y., Gu Q. (2010). Effect of probiotic on growth performance and digestive enzyme activity of arbor acres broilers. Res. Vet. Sci..

[71] Goulet O. (2015). Potential role of the intestinal microbiota in programming health and disease. Nutr. Rev..

[72] Levy M., Blacher E., Elinav E. (2017). Microbiome, metabolites and host immunity. Curr. Opin. Microbiol..

[73] Mohamed T.M., Sun W., Bumbie G.Z., Elokil A.A., Mohammed K.A.F., Zebin R., Hu P., Wu L., Tang Z. (2021). Feeding *Bacillus subtilis* ATCC_19659_ to broiler chickens enhances growth performance and immune function by modulating intestinal morphology and cecum microbiota. Front. Microbiol..

[74] Li Y., Xu Q., Huang Z., Lv L., Liu X., Yin C., Yan H., Yuan J. (2016). Effect of *Bacillus subtilis* CGMCC 1.1086 on the growth performance and intestinal microbiota of broilers. J. Appl. Microbiol..

[75] Wang Y., Sun J., Zhong H., Li N., Xu H., Zhu Q., Liu Y. (2017). Effect of probiotics on the meat flavour and gut microbiota of chicken. Sci. Rep..

[76] Fan Y., Pedersen O. (2021). Gut microbiota in human metabolic health and disease. Nutr. Rev..

[77] Umesaki Y., Setoyama H., Matsumoto S., Imaoka A., Itoh K. (1999). Differential roles of segmented filamentous bacteria and clostridia in development of the intestinal immune system. Infect. Immun..

[78] Rautio M., Eerola E., Visanen-Tunkelrott M., Molitoris D., Lawson P., Collins M.D., Jousimies-Somer H. (2003). Reclassification of *Bacteroides putredinis* (Weinberg et al., 1937) in a New Genus *Alistipes* gen. nov., as *Alistipes putredinis* comb. nov., and Description of *Alistipes finegoldii* sp. nov., from Human Sources. Syst. Appl. Microbiol..

